# Rod and Cone Function in Patients with KCNV2 Retinopathy

**DOI:** 10.1371/journal.pone.0046762

**Published:** 2012-10-15

**Authors:** Ditta Zobor, Susanne Kohl, Bernd Wissinger, Eberhart Zrenner, Herbert Jägle

**Affiliations:** 1 Institute for Ophthalmic Research, University of Tübingen, Tübingen, Germany; 2 Molecular Genetics Laboratory, Institute for Ophthalmic Research, University of Tübingen, Tübingen, Germany; 3 Department of Ophthalmology, University of Regensburg, Regensburg, Germany; Radboud University Nijmegen Medical Centre, The Netherlands

## Abstract

**Background:**

To investigate rod and cone function and disease mechanisms in patients with KCNV2 retinopathy.

**Methodology/Principal Findings:**

Psychophysical examinations as well as detailed electrophysiological examinations with Ganzfeld and multifocal electroretinogram (ERG) were performed to study response dynamics. Additionally, fundus photography, autofluorescence imaging and spectral domain OCTs were carried out for morphological characterization. Molecular genetic analysis revealed compound heterozygosity in five patients and homozygosity for the *KCNV2* gene in one patient. The mutations resulted in complete absence of Kv8.2 subunits in three patients (no protein group, NOP), while the other three patients expressed mutant Kv8.2 subunits resulting in altered Kv2.1/Kv8.2 heteromeric or residual Kv2.1 homomeric potassium channel function (altered protein group, ALP). Although more advanced morphological changes were visible in the NOP group, a clear functional difference between the two groups could not be observed. All patients showed characteristic dynamics of the b-wave intensity-response function, however, scotopic b-wave response amplitudes were within normal limits. We also observed severely reduced oscillatory potentials.

**Conclusions/Significance:**

A specific genotype-phenotype correlation in retinal function could not be demonstrated. *KCNV2* mutations cause a unique form of retinal disorder illustrating the importance of K^+^-channels for the resting potential, activation and deactivation of photoreceptors, while phototransduction remains unchanged. The reduced oscillatory potentials further suggest an altered function of the inner retina. Besides the characteristically steep amplitude-versus-intensity relationship, flicker responses at intermediate frequencies (5–15 Hz) are significantly reduced and shifted in phase.

## Introduction

In 1983 Gouras et al. [1] reported an unusual type of retinal dystrophy, which was associated with characteristic alterations in the rod electroretinogram (ERG). This rare, autosomal recessive condition has been reported in several further studies [2], [3], [4], [5], [6], [7], [8], [9] and was named “cone dystrophy with supernormal rod responses (CDSRR)”. CDSRR is characterized by an early markedly reduced central visual acuity with central scotoma, photophobia, severe color disturbances, and occasionally nystagmus. In contrast to other cone dystrophies, a disease-typical alteration of the rod system could be observed: while rod sensitivity to weak flashes was reduced, an augmented responsiveness to higher levels of flash stimuli could be detected, and implicit times were considerably prolonged [1], [2], [3], [4], [5], [6], [7], [8], [9]. These characteristics were unique for CDSRR, however, the underlying disease mechanism could not be elucidated at that time.

In 2006 Wu et al [10] successfully linked the disorder to chromosome 9p24 and the *KCNV2* gene, which is predominantly expressed in retinal rod and cone photoreceptors [11]. It encodes a member of voltage gated potassium channels (Kv channels), representing a silent subunit (Kv8.2) that is able to assemble with Kv2.1 to form functional heteromeric channels. This results in a shift in the steady-state activation curve of the Kv2.1 channel towards more negative potentials due to a permanent outward K^+^ current, a lower threshold potential for activation, a shortened activation time and slower inactivation kinetics [11], [12], [13]. A mutation in *KCNV2* may thus alter important characteristics of the I_kx_ current that influences the photoreceptor membrane potential. However, the dysfunction and mechanisms that link *KCNV2* mutations with the clinical picture still remain to be elucidated.

Over 50 different mutations in *KCNV2* have been reported so far, mainly small indel mutations or point mutations that constitute protein truncation mutations and amino acid substitutions [10], [14], [15], [16]. Recently, several large deletions within or of the *KCNV2* gene of up to 237 kb in size have been described [16]. Although the genetically detected patients did show altered rod responsiveness, the term “supernormal rod response” was in many cases deceptive, as previously shown [17]. The term “supernormal rod ERG” is a misnomer and most recently, the disorder has been referred to as “KCNV2 retinopathy” [18].

This study employs detailed psychophysical and electrophysiological testing as well as spectral domain optical coherence tomography (OCT) and fundus autofluorescence (FAF) to reveal novel insights into disease-specific functional changes in KCNV2 retinopathy. Additionally, we explore differences of disease specific functional aspects in the phenotype that correlate with the underlying *KCNV2* gene alterations. The genotype of three patients has already been published elsewhere [19], the remaining three patients' genetic findings are presented here for the first time.

## Methods

### Patients

Six otherwise healthy patients of German origin (3 female and 3 male; 2 simplex cases and 2 sibling pairs; mean age: 39 years, range 28–60 years) with previously diagnosed stationary retinal disorder and known mutations in the *KCNV2* gene were examined.

All examinations were carried out after written informed consent and in accordance with the Declaration of Helsinki. The study was approved by the Ethics Committee of the Medical Faculty of University of Tübingen.

### Molecular Genetics

Genomic DNA was extracted from venous EDTA-blood samples according to standard procedures. Genetic testing for point mutations was performed by PCR amplification and subsequent Sanger sequencing of both coding exons and flanking intronic sequences of the *KCNV2* gene, as described previously [16].

Analysis for genomic deletion was investigated by quantitative copy number analyses of the *KCNV2* gene, with realtime PCR employing TaqMan technology or SYBR Green detection assays, as reported earlier [16].

Comparative genome hybridizations (CGH) using a predesigned chromosome 9 specific 385k oligonucleotide array (HG18 CHR9 FT; Roche NimbleGen Inc., Madison, WI) was performed for subject CHRO8.I who had suspected deletions at the *KCNV2* locus (Roche NimbleGen).

The deletion junctions in patients CHRO8.I and RCD307 were determined by long distance PCR amplifications and subsequent Sanger sequencing to define the precise breakpoints.

Independent segregation of the mutations within the families were conducted by Sanger sequencing of PCR amplified genomic DNA for point mutations, and by qPCR in the two families segregating the *KCNV2* gene deletion.

### Clinical Examination

A complete ophthalmological examination was performed including psychophysical tests (Snellen visual acuity, Lanthony Panel D-15 and Nagel anomaloscope color vision tests, visual field and dark adaptation) and an extended electrophysiological protocol (Ganzfeld and multifocal ERG).

### Psychophysical testing

Kinetic 90° and static 30° visual field tests were carried out with an Octopus 900 perimeter (Haag-Streit International, Germany). Dark adaptation curves were measured with a dark adaptometer (Roland Consult GmbH, Brandenburg, Germany) after pupil dilation with Tropicamid. After 3 minutes of bleaching with bright white light (intensity 5.5 log photopic trolands), a staircase procedure was used to estimate detection thresholds over a period of 40 minutes. Thresholds were alternately measured for red (635 nm) and green (565 nm) circular targets, presented 20° nasal of the fovea. Cone and rod thresholds were then determined by a model fit using the equation:
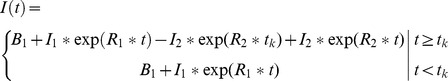
with *t_k_* describing the time to the rod-cone break and *I*1, *R*1 the exponential decay of the cone, *I*2, *R*2 of the rod thresholds. Cone and rod parameters are derived from red and green target functions respectively. For group analysis and for comparison to normals the model was fitted to the raw data of each group of subjects.

### Electrophysiological testing

Ganzfeld and multifocal electroretinograms ERGs were recorded according to the standards of the International Society for Clinical Electrophysiology of Vision (ISCEV) [20], [21]. All tests were performed using DTL electrodes with an Espion E^2^ (Diagnosys LLC) recording device coupled with a ColorDome (Diagnosys LLC) as light source. After 30 minutes of dark adaptation a series of responses to increasing flash intensities (4 ms–0.0001 cd.s/m^2^ to 10 cd.s/m^2^ in 0.5 log unit steps) were recorded and the stimulus-response (S-R) functions modelled using the equation:
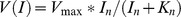
with the saturated b-wave amplitude *V_max_*, the flash intensity *K* required for semi-saturation as a measure of retinal sensitivity and the slope related exponent *n* [22].

Rod response characteristics were estimated from the a-wave by the Hood and Birch (1994) formulation of the Lamb and Pugh model [23] of the biochemical processes involved in the activation of rod phototransduction. The a-wave ensemble was fitted with a computational model describing the response (*P_III_*) as a function of time (*t*) and intensity (*I*):
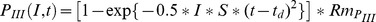
where *Rm_pIII_* is the maximum amplitude, *S* is a sensitivity variable and *t_d_* is a brief delay before the response onset.

*P_III_* was then subtracted from the original ERG waveform to give the *P_II_* response, which is thought to represent mainly the ON-bipolar cell response, but also the postreceptoral activity in other second- and third-order retinal neurons. The relation between flash intensity and the delay between stimulus onset and reaching a given arbitrary criterion voltage of the *P_II_* component was then plotted on a log-log coordinate and the slope of this function was calculated. The voltage criterion chosen in this study was 50 µV.

Finally, dark-adapted responses to a series of blue flicker (LED 470 nm) with an intensity of 0.03 cd.s/m^2^ and frequencies between 5 and 30 Hz were recorded to isolate temporal retinal characteristics of the rod system [24].

The light-adapted protocol (10 min of light adaptation to a background luminance of 30 cd/m^2^) included a single flash cone stimulus and a 30 Hz flicker (both: 4 ms, 3.0 cd.s/m^2^). In addition, responses to a series of flicker white stimuli of 3.0 cd.s/m^2^ with increasing frequency from 5 to 45 Hz were included to investigate possible alterations in the temporal resolution of the cone retinal pathway.

Multifocal ERG (mfERG) was performed with a VERIS System (Version 5.1) using a Grass amplifier (model 12, Quincy, USA). The stimulus, consisting of 61 scaled hexagonal elements covering a central visual field of 60×55°, was presented on a 19″ monitor at a frame rate of 75 Hz at a distance of 32 cm from the subject's eyes. The same DTL electrodes as those for the Ganzfeld recordings were used. Responses were amplified (200 000×), bandpass-filtered (10–100 Hz), and analysed according to ring averages.

### Morphological testing

Color and infrared fundus photography, autofluorescence (FAF) and spectral domain OCT recordings (Heidelberg Engineering GmbH, Germany) were performed.

## Results

### Molecular Genetic Findings

**Figure 1:**
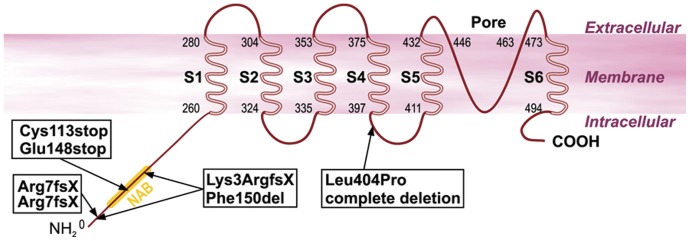
Structure of the Kv8.2 potassium channel and mutation sites detected in our patients. The mutation pairs in each box represent the genetic findings of each patient. The genetic findings of the two sibling pairs are shown once, since the siblings share the same mutation constellation.

Mutation screening and segregation analysis led to the identification of mutations in the *KCNV2* gene in our patients. The genotypes of the six patients are listed in **Table 1**. and the mutation localization is shown in **Figure 1**. We observed compound heterozygous mutations in the two sib pairs (CHRO8.I and CHRO8.II, and BD27.I and BD27.II): both patients from family CHRO8 carried two compound heterozygous nonsense mutations p.Cys113stop and p.Glu148stop, while both patients of family BD27 were compound heterozygous for a complete deletion of the *KCNV2* gene and a missense mutation p.Leu404Pro located in the linker between transmembrane domains S4 and S5. The simplex subject BCM5 harboured two compound heterozygous small deletions: c.8_11del and c.447_449del. The c.8_11del mutation created a frame-shift at the very beginning of the *KCNV2* polypeptide, resulting in a premature stop codon and a severely altered and truncated protein (p.Lys3ArgfsX95). The other deletion only resulted in the loss of a single phenylalanine at position 150 (p.Phe150del) within the NAB domain. The last patient RCD307 was homozygous for another large deletion spanning from exon 1 into the 3′UTR. All mutations, except for the missense mutation p.Leu404Pro and the single amino acid deletion p.Phe150del (see **Table 1**.), are expected to result in the complete loss of Kv8.2. Consequently the siblings BD27.I and BD27.II, and patient RCD307 most likely did not express any KV8.2 gene product, causing an altered subunit composition of the respective Kv-channel and an altered or lost potassium channel function. Previous clinical data have indicated that both the complete absence of Kv8.2 (where Kv2.1 was unaffected) and its altered forms result in CDSRR [10], which suggests that the special constellation of Kv2.1/Kv8.2 heteromeric channels are essential for functionality in the photoreceptor cells. To examine whether these genotype differences are also evident in the phenotype, we divided the six patients into two groups: group 1, NOP (no protein), included the three patients with a complete absence of Kv8.2, and group 2, ALP (altered protein) with three patients with mutant Kv8.2 subunits.

**Table 1:** Clinical and genetic findings of the six patients.

Patient ID	Gender	Age	VA (RE/LE)	Refraction	Night blindness	Color vision	Nystagmus	Strabismus	Alteration nucleotide sequence	Alteration polypeptide	Allele status
**BD27.I**	M	36	0.125	−2.0/−1.0×145°	no	achromat	no	no	c.339C>A	p.Cys113X	heterozygous
			0.1	−1.0					c.442G>T	p.Glu148X	
**BD27.II**	F	40	0.04	−3.5/−2.25×174°	no	achromat	yes	yes	c.339C>A	p.Cys113X	heterozygous
			0.05	n.A.					c.442G>T	p.Glu148X	
RCD307*	M	60	0.16	−3.5/−1.25×0°	yes	protanomalous	no	no	c.19_1356+9571 delinsCATTTG	Arg7fs	homozygous
			0.1	−1.0/−1.0×0°							
CHRO8.I*	M	37	0.1	−8.75/−2.0×18°	no	achromat	yes	yes	g.2657638_2737340del	Deletion	heterozygous
			0.1	−8.25/−2.0×141°					c.1211T>C	p.Leu404Pro	
CHRO8.II*	F	35	0.1	−4.5/−4.0×0°	no	achromat	no	yes	g.2657638_2737340del	Deletion	heterozygous
			0.2	−3.5/−4.0×0°					c.1211T>C	p.Leu404Pro	
**BCM5**	M	28	0.16	−5.0/−1.5×2°	no	achromat	no	yes	c.8_11del4	p.Lys3ArgfsX29	heterozygous
			0.16	−3.5/−1.0×6°					c.447_449del3	p.Phe150del	

The upper three patients show a complete absence of Kv8.2 due to large deletions or protein truncating mutations of *KCNV2* and so represent the NOP group. The lower three patients represent the ALP group with altered Kv8.2 subunits due to *KCNV2* mutations. The BD27.I and BD27.II are brother and sister of one family, so are CHRO8.I and CHRO.II of another family. Each case shows an autosomal recessive inheritance. A considerable difference between the NOP and ALP group cannot be observed. However, there is a tendency for a slightly better visual acuity (VA) and higher myopia in the ALP group (lower three patients). Interestingly, night blindness, subjective disease progression and a severe protanomaly are present in the only homozygous patient (RCD307), while other patients reveal unchanged visual function over disease duration, no night blindness and color disturbances consistent with a rod dominated function. (Patients marked with * have been published in Wissinger et al. [19]).

### Clinical Findings

Clinical findings are summarized in **Table 1**. All patients reported an early onset of their visual symptoms without any progression or change over the years. Only the oldest patient (RCD307) reported a slight reduction of his visual acuity in the last three years. Marked photophobia and a prolonged light adaptation time were evident in every case. Only the oldest patient complained of nyctalopia, other subjects denied having difficulty with night vision. All patients were myopic with variable degrees of astigmatism; patients of the ALP group showed a slight tendency for higher myopia and astigmatism. Four of six patients had undergone strabological surgery in childhood and two of them had suffered additionally from infantile nystagmus.

### Psychophysics

All subjects presented with reduced central visual acuity (mean VA (logMAR): 0.97±0.2 SD). There was a slight tendency to poorer VA in the NOP group (1.06±0.23 SD) compared the ALP group (0.88±0.13 SD). Color vision testing using the Lanthony D-15 Panel desaturated and saturated tests and with fixation with the preferred retinal locus (PRL) showed severe color confusions in all patients predominantly along the scotopic or red-green axis with relative sparing of the tritan axis. The Rayleigh anomaloscope matches, presented eccentrically at the PRL, were consistent with a rather rod dominated function in five of six patients. Only one patient's (RCD307) results suggested protanopia. Perimetric results showed nearly normal outer boundaries of the visual field in all cases. Static perimetry results revealed relative and absolute defects in the central 30° area, being more pronounced in the NOP than the ALP group.

**Figure 2:**
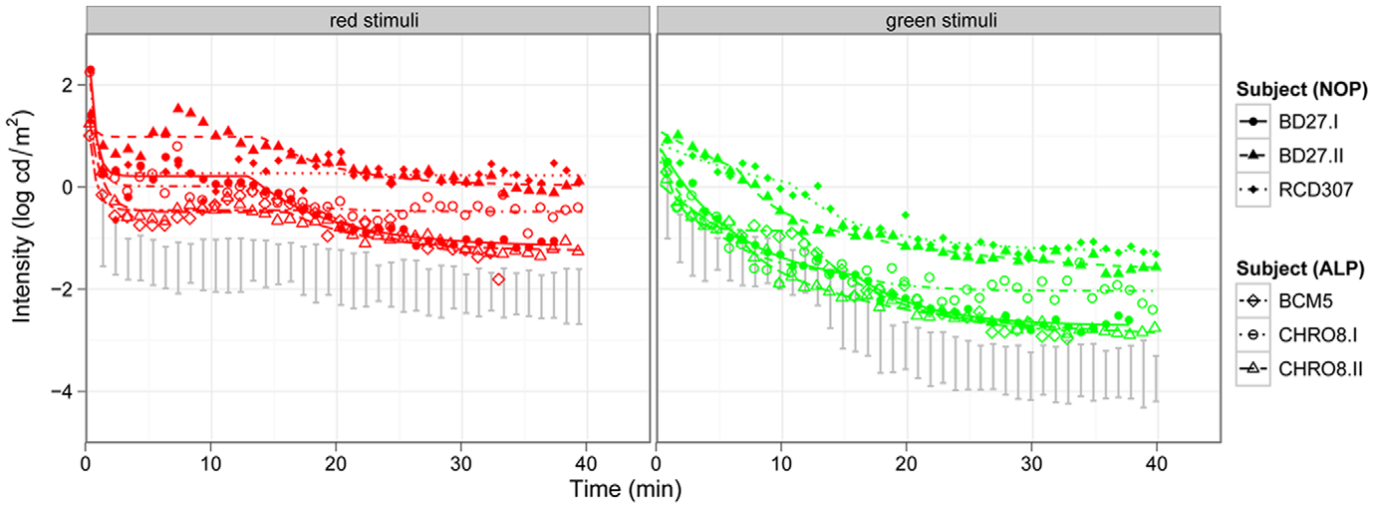
Dark adaptation curves of the patients of both groups compared to normal subjects. The left panel shows the cone (red stimuli), the right panel the rod function (green stimuli) of the NOP and ALP groups (the 95% confidence band of normal subjects is marked with grey). The threshold elevation for both target colors is biggest for two patients of the NOP group.

To test cone and rod function loss we measured dark adaptation thresholds for red and green targets. (**Figure 2**). All patients showed significantly elevated thresholds for red and green stimuli, although the elevation was more pronounced in the NOP group with a final rod threshold of −1.9 log cd/m^2^ and a cone threshold of 0.4 log cd/m^2^ compared to −2.6 log cd/m^2^ and −0.3 log cd/m^2^ for the rod and cone threshold in the ALP group. Thresholds estimated for normals were −1.5 log cd/m^2^ and −3.7 log cd/m^2^ for red and green stimuli, respectively. For green stimuli the rod-cone break was normal (11.0 min for the NOP and ALP group, normal: 10.7 min) and for red stimuli the rod-cone break tended to appear earlier (after 15.6 and 15.9 min for NOP and ALP respectively, normal: 16.9 min).

### Electrophysiology

**Figure 3:**
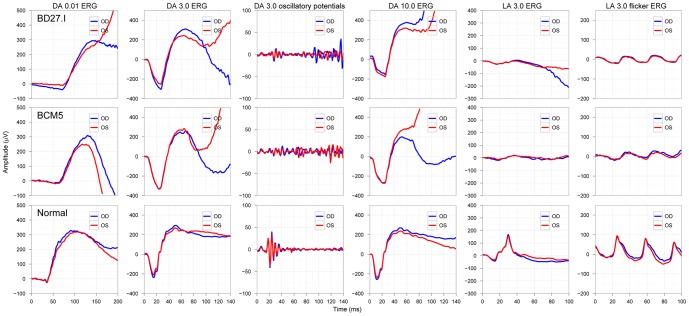
ISCEV standard Ganzfeld ERG responses of a representative patient of each group compared to a normal subject. Patient BD27.I in the upper panels represents the NOP group, while the result of patient BCM5 in the middle panels is an example for the ALP group. The normal subject is represented in the bottom panels. The patients' findings show the characteristic features of the scotopic ERG (dark-adapted (DA)) and the small photopic responses (light adapted (LA). Notice the missing oscillatory potentials on the rising b-wave in patients. (Color coding in each panel: red curves show results of the right eye (OD), blue curves show results of the left eye (OS)).

**Figure 4:**
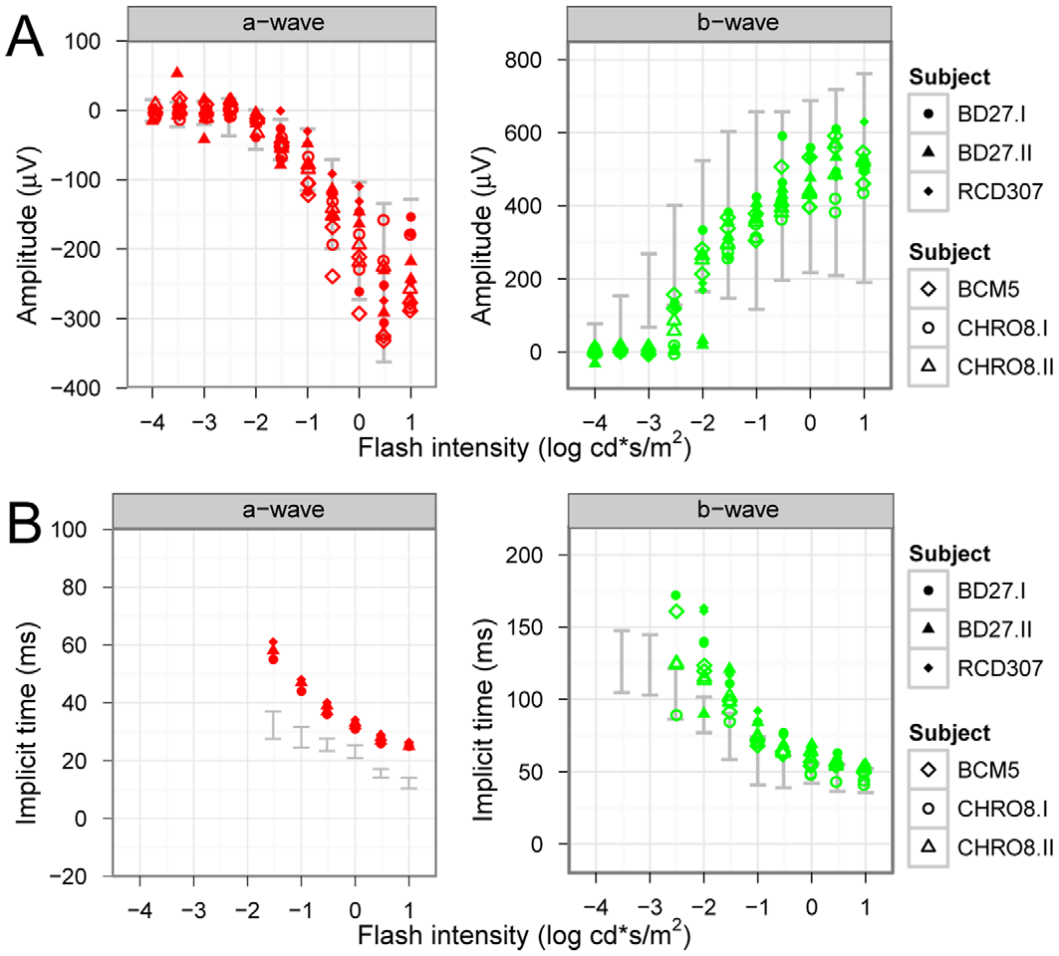
Intensity-response function kinetics under scotopic conditions. The single flash response a- and b-wave amplitudes (A) and implicit times (B) to increasing stimulus intensities are presented for both groups (upper subjects: NOP group, lower subjects: ALP group; the 95% confidence band of normal subjects is marked with grey). While the a-wave amplitude slowly and continuously increases, the b-wave amplitude stays low until the flash intensity reaches −2.5 log cd*s/m^2^. While peak implicit times of the a-wave responses are prolonged for all flash intensities, the implicit times of the b-wave approaches the normal range with increasing flash intensity.

Basic clinical investigation included the Ganzfeld ERG according to the ISCEV standard, for which all patients showed the previously described characteristic responses (see **Figure 3** for typical results of a patient from the NOP and ALP group). Most interestingly, oscillatory potentials (OPs) were almost completely absent in the patients' ERG recordings. **Figure 4** shows for each subject the amplitudes and implicit times of the a- and b-waves for the scotopic response series recordings. Typical low or undetectable response amplitudes to weak flashes were evident, with markedly delayed implicit times of the a- and b-wave component. There was also an abrupt increase in amplitude with increasing flash intensity accompanied by a normalization of b-wave implicit times. While mean saturation amplitudes *V_max_* of the b-wave model fit were similar for both groups and normals (526 µV for normals, 528 and 493 µV for the NOP and ALP groups, respectively) the intensity *K* at semi-saturation was significantly shifted to higher intensities (−2.5, −1.7 and −1.7 log cd.s/m^2^ for normals, NOP and ALP, respectively). Additionally, the peak a-wave amplitude was normal within the entire stimulus intensity range, but the peak implicit times were prolonged for each stimulus step.

**Figure 5:**
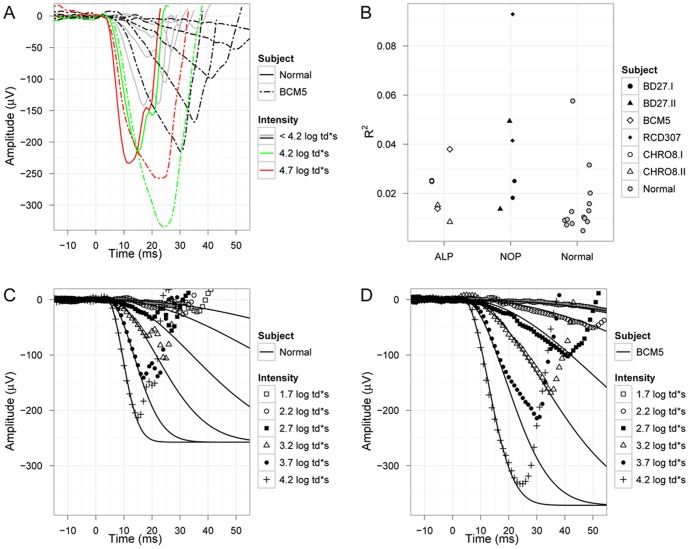
Analysis of the scotopic a-wave (*P_III_* response). (A) Scotopic response a-waves to increasing flash intensities show continuous increasing response amplitudes in the normal subject (full lines). The patient's responses (chain lines) also show continuous increasing amplitudes up to a flash intensity of 4.2 log td*s (green color coding), but a lower response amplitude to the flash with highest intensity (red color coding). Therefore responses to highest flash intensity were excluded from a-wave analysis. Interestingly, while R^2^ of the fits in normal subjects was above 0.02 in 2 eyes only, it was higher in 7 of the patient eyes (B). The difference in goodness of the fit is seen in the normal subject (C) and in the representative patient BCM5 (D).

In this study the rod a-wave showed three interesting features (**Figure 5**.): First, in some patients the a-wave of the response to the highest intensity stimulus (4.7 log td*s) was smaller than that to the 4.2 log td*s stimulus. This is depicted for one subject in **Figure 5A**. While R^2^ of the fits in normal subjects was above 0.02 in only two eyes, it was higher in seven of the patient eyes (**Figure 5B**). The difference in goodness of the fit is seen in the normal subject in **Figure 5C** and in the representative patient BCM5 in **Figure 5D**.

Second, the latency of the negative deflection from baseline appeared with normal delay (average of 4.1±0.92 ms and 4.3±0.35 ms for patients and normals respectively).

And third, while the maximum response amplitude Rm_PIII_ estimated from the model fit was not different from that of normals (on average 241±71 µV and 270±83 µV for patients and normals respectively), the sensitivity parameter S was significantly lower in patients (0.73±0.38 SD) than in normals (1.14±0.36 SD).

**Figure 6:**
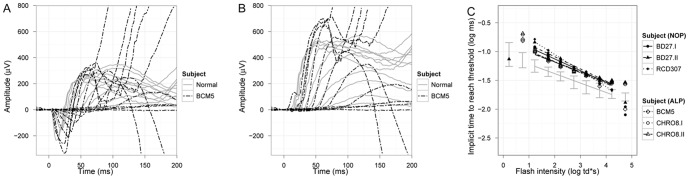
Analysis of the *P_II_* response. Overlaid representative scotopic waveforms of a normal subject (grey lines) and patient BCM5 (chain lines) and the extracted P_II_ components (B) are presented. The delay in implicit times to reach a threshold amplitude of 50 µV at each intensity level is plotted in a log-log diagram both for the NOP group and ALP group (C).

*P_II_* responses were calculated by removal of the fitted *P_III_* (**Figure 6**.). The latency at which *P_II_* reaches 50 µV is plotted as a function of stimulus intensity on log-log coordinates in **Figure 6C**. In the normal retina we found a slope of −0.18±0.014 SD. In patients with *KCNV2* mutations the mean slopes of regression lines were not significantly different from each other or from the normal mean slope (−0.24±0.065 SD and −0.20±0.038 SD for the NOP and ALP group, respectively), however, a similar and clear shift either representing a response delay or a horizontal shift to higher intensities of approx. 1 log unit was observed in both groups. Latter was consistent with the psychophysically (dark adaptation) estimated rod threshold elevation of approx. 1 log unit.

**Figure 7:**
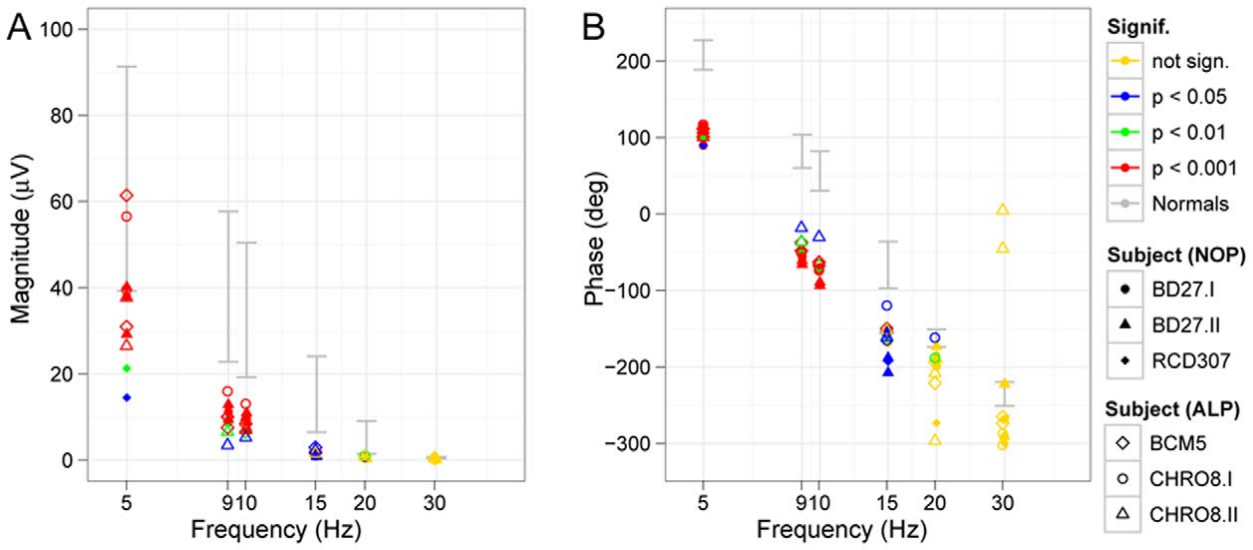
Scotopic flicker series. Magnitude (A) and phase (B) of scotopic flicker responses to stimuli of increasing flicker frequency are presented. Significance levels are color-coded in each figure, the 95% confidence band of normal subjects are marked with grey. The amplitudes of the patients are below normal and not significant for stimuli faster than 15 Hz. The phase shift, however, seems to be independent from flicker frequency.

We additionally recorded responses to various flicker frequencies under scotopic conditions (**Figure 7**). Magnitude (**Figure 7A**) and phase (**Figure 7B**) of the responses to increasing flicker frequencies are demonstrated. The individual data under scotopic conditions showed reduced magnitudes and a phase difference, which was independent from flicker frequency thus suggesting either a rather small constant prolongation of rod photoreceptor recovery or may result from the sensitivity reduction to the flash strength.

**Figure 8:**
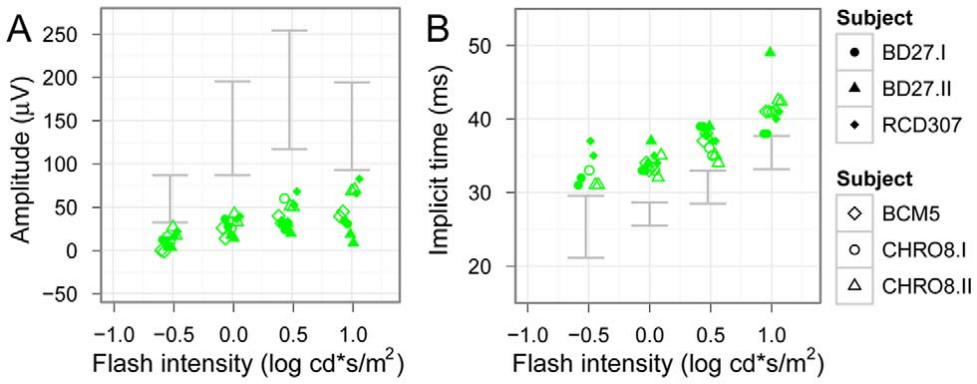
Intensity-response function kinetics under photopic conditions. Photopic response b-wave amplitudes are shown in (A) and implicit times in (B), the 95% confidence band of normal subjects is marked with grey. The photopic hill phenomenon can be observed in normals, however, this phenomenon seems to be missing in patients. Implicit times in both patient groups are moderately delayed (upper subjects: NOP group, lower subjects: ALP group).

**Figure 9:**
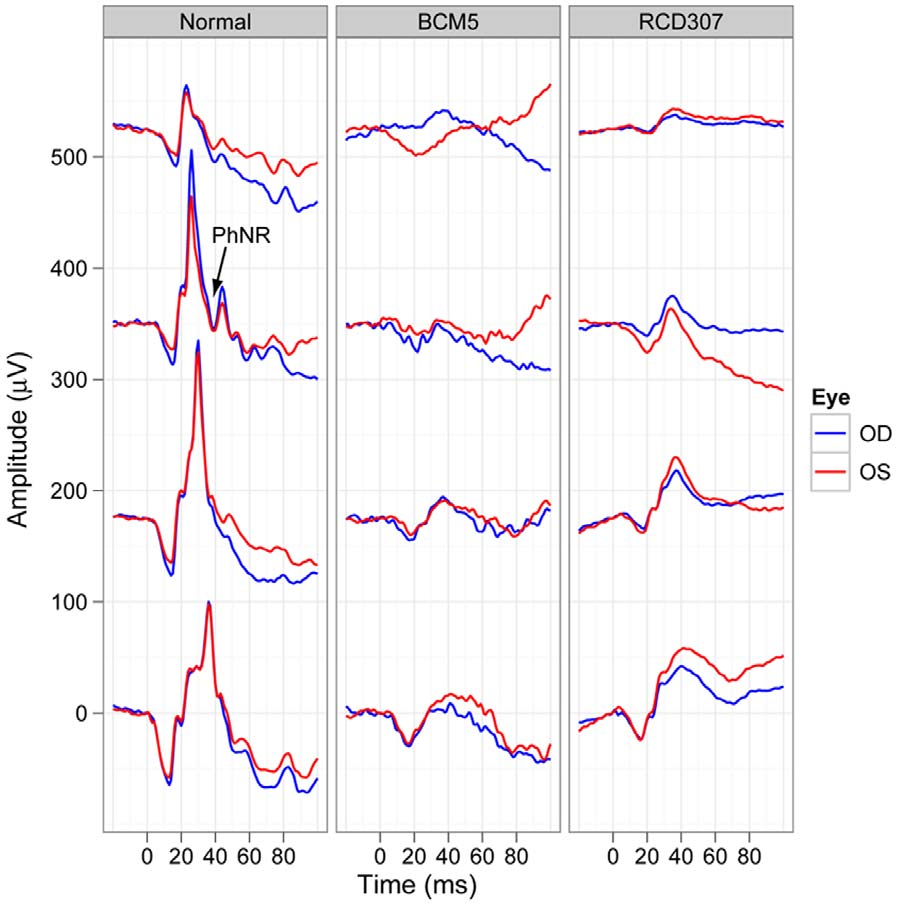
Photopic negative response (PhNR). Photopic single flash response waveforms illustrating the photopic negative response (PhNR) at increasing stimulus intensities (from top to the bottom in each panel) are shown in a normal subject (on the left) and the waveforms of two representative patients of the ALP (patient BCM5 in the middle) and NOP group (patient RCD307 on the right). Notice, that patients lack the PhNR in the given intensity range.

Cone responses recorded under photopic conditions showed a marked suppression of response amplitudes, which remained diminished even at the higher flash intensities (**Figure 8**.). Additionally, the amplitudes did not show a photopic hill phenomenon. Implicit times were markedly prolonged for each stimulus step. The photopic negative responses (PhNR) were almost undetectable in patients with *KCNV2* mutations, as depicted in **Figure 9**.

**Figure 10:**
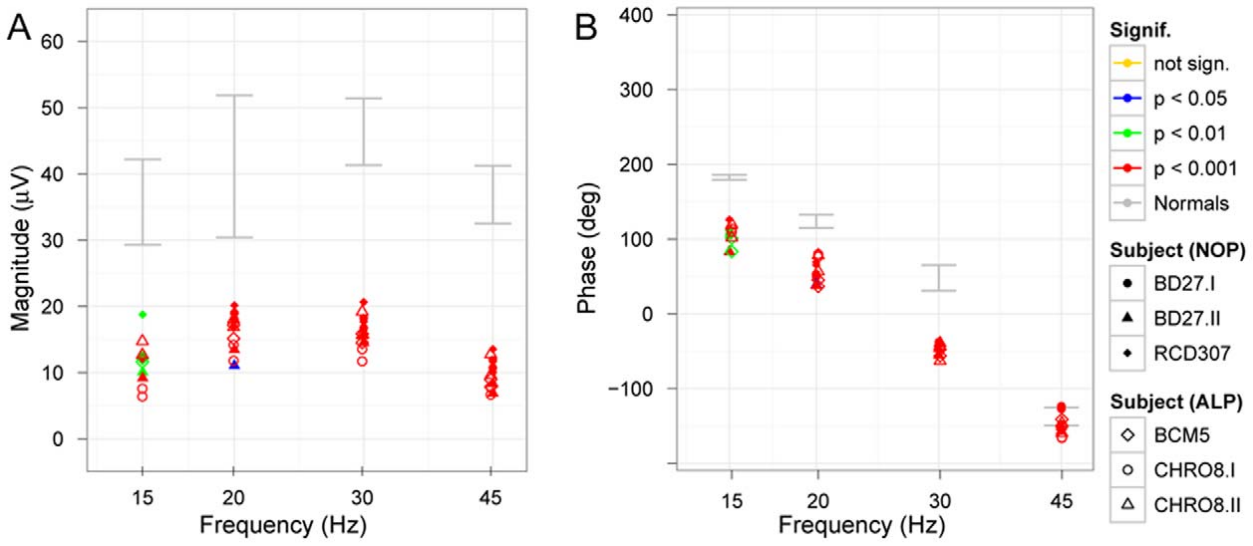
Photopic flicker series. Magnitude (A) and phase (B) of photopic flicker responses in a flicker series show similar frequency dependence but lower magnitude in patients accompanied by a phase shift. Significance levels are color-coded in each figure, the 95% confidence band of normal subjects is marked with grey.

As found for scotopic conditions, the photopic responses to stimuli of increasing frequency (**Figure 10**.) showed a similar dependency but also lower amplitudes and a shift in phase. Interestingly, even at the highest frequency of 45 Hz the response waveform was still significant with an almost normal phase.

**Figure 11:**
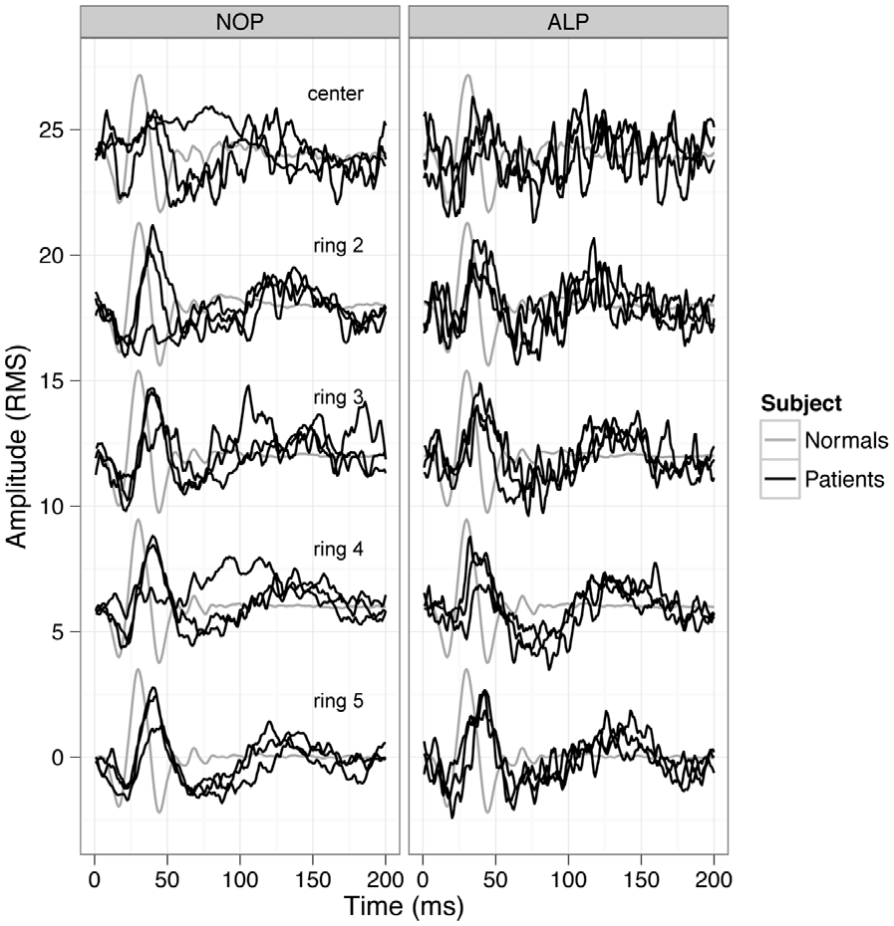
MfERG results. Individual mfERG response waveforms for the subjects of each group (NOP group on the left, ALP group on the right) compared to normal subjects waveforms (black curves represent the responses of the patients, grey curves show normal responses). Responses of patients are reduced and delayed in both groups, more pronounced in the central rings. Interestingly, in the ALP group the central 2 rings tend to show more preserved responses. This could be in relation with the slightly better VA of the ALP group.

Finally, multifocal ERGs showed reduced amplitudes and delayed implicit times in every ring (**Figure 11**.) being more distinct in the central 2 rings. In the outer rings more preserved responses could be obtained. The dysfunction was more sharply limited to the central two rings in the NOP group, which was not observed that clearly in the ALP group. This could be related to the slightly better VA of the ALP group.

### Morphology

**Figure 12:**
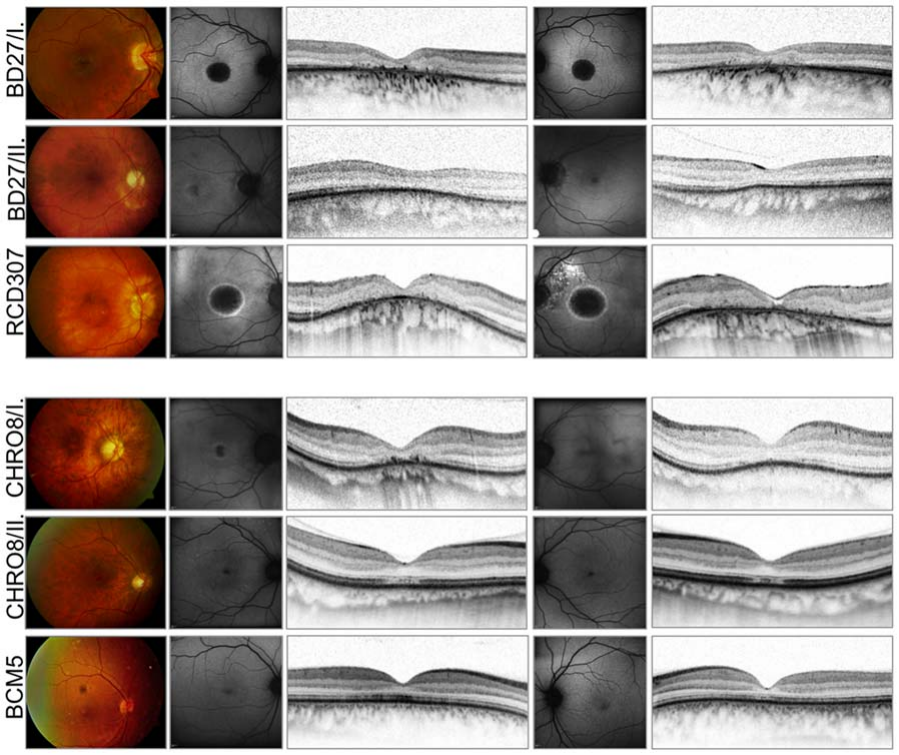
Morphological findings. Fundus photographs of the right eye, FAF and spectral domain OCT images of both eyes of six patients illustrating the variability and extent of foveal changes (NOP group: upper three patients, ALP group: lower three patients). Notice the age-related changes, epiretinal gliosis and macular hole formation on the left eye of the RCD307 patient.

Fundus photographs, FAF images and OCT scans for each subject can be seen in **Figure 12**. There was a range of macular appearances including discrete disturbances of the retinal pigment epithelium (RPE) and bull's eye maculopathy. Patients with a complete absence of Kv8.2 (NOP group) showed more pronounced changes in the macular area, the mean central retinal thickness was 103.5 µm for the NOP, and 135.1 µm for the ALP group, respectively. In FAF imaging mild RPE-alterations were present as small areas with decreased autofluorescence. In contrast, marked RPE-atrophies were seen as sharply demarcated areas of absent autofluorescence surrounded by a ring of increased signal. The oldest patient (RCD307) revealed additional RPE-defects of the posterior pole and epiretinal gliosis. The OCT images also demonstrated the variety of morphological findings. In the milder cases –mainly in the ALP group- OCT revealed a thinner photoreceptor layer (PRL) in the foveal area, there was no disruption in the inner segment/outer segment (IS/OS) border. In the more severe cases (NOP group) the PRL was missing, the IS/OS border was diminished and an increased backscatter from the choroid was observed due to RPE-atrophy. In one case additional granular echoes were present due to deposits on the fundus of patient BD27.I. Based on the SD-OCT volume scans detected from the central 30°×15° retinal area the peripheral outer retinal structure was well preserved in every patient.

## Discussion

This study describes the genotype and phenotype of six patients with a retinal dystrophy due to changes in the *KCNV2* gene. In three patients the mutations resulted in a complete absence of Kv8.2 encoded by the *KCNV2* gene (NOP group). In the other three patients heterozygous mutations of the first allele resulted in a lack of protein product, mutations of the second allele led to mutant subunits with presumably remaining pore function (ALP group).

On the morphological level, macular pathology varied from mild RPE disturbances to large atrophic areas, while the periphery was normal in every case. These findings are consistent with the results described by Robson et al. and Sergouniotis et al. [18], It is interesting to see that the changes are strictly limited to the central macular area in every case, while the specific functional changes of this retinal disorder affect rod-rich mid periphery as well. The question of why cones degenerate and rods keep at least their morphological integrity still remains to be unraveled, but the distribution of Müller cells in the retina (i.e. absence in the fovea) could be one important factor, since their regulatory and buffering effect on the extracellular K^+^ is missing in the cone-rich area, making cones more vulnerable.

Although there was a tendency for a more pronounced macular lesion (seen in the OCT and FAF imaging), higher myopia and more elevated dark adaptation thresholds in the NOP group, no clear-cut line can be drawn between the two groups. Interestingly, the only homozygous patient (RCD307) seemed to differ in a few aspects from the heterozygous patients: night blindness, protanomaly and reported progression were present only in his case. There was no family history of color disturbances in this subject and the further genetic analysis of the L-M pigment genes showed an intact OPN1MW/OPN1LW gene cluster [25], [26], indicating a severe protanomaly rather than protanopia. Furthermore, his morphological results revealed more distinct changes of the fovea resembling a macular hole on the left eye. However, the additional RPE-defects of the posterior pole, the macular hole formation and the accompanying epiretinal gliosis could be due to age related changes, explaining the decreasing visual acuity observed by the 60-year-old patient. However, the investigated cohort is small, further large studies are necessary to better highlight the correlations between genotype and phenotype.

Several studies exist on the electrophysiological characteristics of KCNV2 retinopathy. [1], [4], [6], [17], [27]. Nevertheless, there are still several aspects of this special retinal disorder, for which an explanation is needed

The lack of Kv8.2 or the presence of mutant subunits eliminates the functional characteristics of Kv2.1/Kv8.2 heteromers leading to a retinal disorder. Only the intact heteromers have the essential specifics to function as a high-pass amplifier and so regulate photoreceptor responses to light flashes. Kv2.1 can form homomeric channels, but without the intact Kv8.2 subunits they activate more slowly and inactivate faster, whereas the voltage dependence of the steady-state inactivation remains unchanged [11], [12].

The absence of intact Kv8.2 subunits therefore leads to a positive shift of the steady-state membrane potential, decreasing the dark current and elevating intracellular K^+^ level. Kv2.1 channels alone do not produce a permanent outward K^+^ current, which also affects the K^+^ homeostasis of the photoreceptors. While restoring the integrity of the K^+^ levels, secondary mechanisms can also lead to a small drop of intracellular Ca^2+^ levels, probably due to altered/enhanced function of the Na^+^/Ca2^+^-K^+^ exchanger. The small drop of cytoplasmic Ca^2+^, however, can result in increasing cGMP levels due to disinhibiting the activated guanylate-cyclase and finally increases the number of open cyclic nucleotide-gated channels. This shift to a more depolarized state in the dark may also have consequences for recovery: for very brief light flashes the membrane potential may remain below the critical limit of −50 mV before the hyperpolarization-activated cyclic nucleotide-gated channels (HCN channels) become activated [28], thus leading to a prolonged hyperpolarization phase. These mechanisms are well reflected in the electrophysiological findings observed in patients with KCNV2 retinopathy [4], [6], [17]. The responses are characteristically undetectable or markedly reduced with delayed implicit times for dimmer stimuli and there is an abrupt rise in amplitudes and shortening of implicit time with increasing stimulus intensity. The intensity *K* at semi-saturation is significantly shifted to higher intensities, the estimated difference in our cohort is around 1 log cd.s/m^2^. This shift correlates with the threshold elevation during dark adaptometry (for rod thresholds approximately 1 log unit elevation). However, most of our patients do not suffer from nyctalopia, confirming other reports of this inconsistency between subjective and objective light sensation [4], [23].

Our results are in accordance with previous reports and also confirm that “supernormal rod responses” in the ERG - believed to be characteristic for this rare condition - often seem to be missing, as responses, even to high intensity flashes, stay within normal limits in many cases [16], [17]. The dynamics of the b-wave intensity-response function is a more constant feature.

In addition, our detailed electrophysiological data show other specific features. The initial phase or leading edge of scotopic response waveforms reflects the activity of photoreceptor cells and arises from light-evoked closure of Na^+^ channels along the plasma membrane of the outer segments. Based on the model fits to our electrophysiological data we conclude that phototransduction activation in this retinal dystrophy is normal, since the onset of the deflection from baseline appears with normal delay. While the maximum amplitude Rm_PIII_ was within normal limits, the sensitivity parameter S was significantly lower. Similar results were reported for the patient described by Tanimoto et al. [29]. On the contrary, Hood et al. [4] found essentially normal sensitivity S and a slightly lower Rm_PIII_, the maximum amplitude, for the rods. However, our patients were included in the study based on confirmed alterations of *KCNV2*, while patients in the study of Hood et al. were chosen on a clinical-electrophysiological basis (i.e. retinal dystrophy with supernormal rod responses). While higher Rm_PIII_ might be explained by an overshoot due to delayed HCN channel activation, lower sensitivity may be related to higher cation channel sensitivity to cGMP due to lower Ca^2+^ levels.

There is also a delayed postreceptoral response, which seems independent from flicker frequency. The delay of the emerging b-wave may have two different origins. Firstly, voltage dependent transmitter release may be delayed due to a small prolongation in reaching hyperpolarization. Secondly, a delayed HCN channel activation and depolarization of the photoreceptor might result in an overshoot of the response of the downstream neuron. Such overshoot would not be associated with changes in the G-protein activation cascade of the bipolar cell, which can be assessed with the *P_II_* response analysis. In our *KCNV2* patients the kinetics of the ON-bipolar cell G-protein cascade seems to be normal, however, the cascade is activated with a clear delay. In the study of Robson et al. [17] including 25 patients with the characteristic scotopic b-wave signs, this delay was seen for the ON-responses and particularly for the OFF-responses as well. A further interesting finding was the marked reduction or even absence of the oscillatory potentials, either suggesting an altered function of the inner retina (i.e. amacrine or interplexiform cells) or more likely a significantly reduced cone function [28].

Moreover, we studied the temporal characteristics of the retina for the first time in this specific retinal disorder. Repetitive stimulation is very demanding for the metabolic process in neurons and changes in the temporal dynamics (e.g. in channelopathies) are evident in flicker ERGs [30]. The impairment of temporal response characteristics can occur due to photoreceptor disturbances as well as postsynaptic mechanisms. Kv2.1/Kv8.2 heteromeric channels contribute to the generation of the K^+^ current responsible for the dynamic signal amplification of photoreceptors. The hyperpolarizing overshoot in response to rapid onset illumination has an important role in increasing the sensitivity to fast changes of illumination. Altered Kv2.1/Kv8.2 heteromers lose their ability to function as a high-pass amplifier, which explains the altered temporal characteristics observed in our patients (i.e. there is a constant prolongation of photoreceptor recovery time). Interestingly, even at the highest frequency of 45 Hz the response waveform was still significant with an almost normal phase, which indicates that even though we find clearly reduced amplitudes and a phase shift due to cone dystrophy, the temporal dynamics seem to be almost normal. There is little known about critical flicker fusion in retinal dystrophies in humans, but it has been shown in RCS rats that with progression of the degeneration the amplitude for higher frequency waveforms declines and the critical flicker fusion frequency is shifted to lower frequencies [31]. However, Kv2.1/Kv8.2 heteromeric channels are not the only components of the outward K^+^ current in photoreceptors, although they are essential for their functional properties.

These special electrophysiological features are considered to be strictly associated with *KCNV2* mutations, but recently Thompson et al. [32] have shown somewhat similar changes of the dark-adapted electroretinogram in patients with *KCNJ10* mutations. The K^+^ channel expressed by the *KCNJ10* gene (Kir4.1) has previously been recognized as pathogenic in man, causing a constellation of symptoms, including epilepsy, ataxia, sensorineural deafness and a renal tubulopathy (EAST syndrome). Kir4.1 constitutes the primary inward rectifying potassium channel of retinal Müller cells [33] and is responsible for the regulation of extracellular K^+^. Thompson et al. nicely demonstrated similar dynamics of scotopic intensity-response function in two of four patients. ERGs to dimmer flash stimuli showed a delay of up to 20 ms before the onset of the b-wave, and with increasing intensity a sudden elevation of amplitudes and normal implicit times could be detected. These similarities of the scotopic ERG could be explained with an altered sensitivity at the synapse between rod and ON-bipolar cells due to mutations in *KCNJ10*. Photopic ERGs of all patients showed reduced amplitudes of the photopic negative response (PhNR) and showed a delay in b-wave time to peak, but the photopic hill was preserved. However, these patients did not develop a cone dystrophy, since mutations in *KCNJ10* primarily affect the Müller cell functions, while mutations in *KCNV2* lead to disturbed functional integrity of the photoreceptors and probably impair their postreceptoral signaling.

KCNV2 retinopathy is considered a very rare retinal disorder associated with high but often normal mixed rod-cone response amplitudes, a marked prolongation of b-wave implicit times and a delayed, almost sudden, steep amplitude-versus-intensity relationship under scotopic conditions. Furthermore, while rod phototransduction is intact, there is a constant delay of the responses, which suggests changes in the synapse or in postreceptoral signaling pathway. Inner retinal involvement is also probable, since oscillatory potentials are almost absent. These findings are diagnostic and are exclusively linked to *KCNV2* mutations.
